# The porcine corneal surface bacterial microbiome: A distinctive niche within the ocular surface

**DOI:** 10.1371/journal.pone.0247392

**Published:** 2021-02-19

**Authors:** Marina L. Leis, Gabriela M. Madruga, Matheus O. Costa

**Affiliations:** 1 Veterinary Clinical Sciences, College of Veterinary Medicine, University of Minnesota, Minneapolis, MN, United States of America; 2 Small Animal Clinical Sciences, Western College of Veterinary Medicine, University of Saskatchewan, Saskatoon, SK, Canada; 3 State University of São Paulo, São Paulo, Brazil; 4 Large Animal Clinical Sciences, Western College of Veterinary Medicine, University of Saskatchewan, Saskatoon, SK, Canada; 5 Department of Population Health, Faculty of Veterinary Medicine, Utrecht University, Utrecht, The Netherlands; University of Minnesota Twin Cities, UNITED STATES

## Abstract

**Purpose:**

The ocular surface microbiome has been described as paucibacterial. Until now, studies investigating the bacterial community associated with the ocular surface through high-throughput sequencing have focused on the conjunctiva. Conjunctival samples are thought to reflect and be representative of the microbiome residing on the ocular surface, including the cornea. Here, we hypothesized that the bacterial community associated with the corneal surface was different from those of the inferonasal and superotemporal conjunctival fornices, and from the tear film.

**Methods:**

Both eyes from 15 healthy piglets were sampled using swabs (inferonasal fornix, superotemporal fornix, and corneal surface, *n* = 30 each) and Schirmer tear test strips (STT, *n* = 30). Negative sampling controls (swabs and STT, *n* = 2 each) and extraction controls (*n* = 4) were included. Total DNA was extracted and high-throughput sequencing targeting the 16S rRNA gene was performed. Bioinformatic analyses included multiple contamination-controlling steps.

**Results:**

Corneal surface samples had a significantly lower number of taxa detected (*P*<0.01) and were compositionally different from all other sample types (Bray-Curtis dissimilarity, *P*<0.04). It also harbored higher levels of Proteobacteria (*P*<0.05), specifically *Brevundimonas* spp. (4.1-fold) and *Paracoccus* spp. (3.4-fold) than other sample types. Negative control STT strip samples yielded the highest amount of 16S rRNA gene copies across all sample types (*P*<0.05).

**Conclusions:**

Our data suggests that the corneal surface provides a distinct environmental niche within the ocular surface, leading to a bacterial community compositionally different from all other sample types.

## Introduction

The conjunctiva, together with the cornea, limbus and tear film form the ocular surface (OS). The OS is the interface between the eye and the external environment, providing an anatomic, physiologic, and immunologic barrier against various challenges such as microorganisms, changes in temperature and humidity, and other hostile factors [1].

Intriguingly but expectedly, initial metagenomics studies suggested that the OS harbors a very low number of microorganisms when compared to other mucosal sites [2, 3]. This is likely due to a combination of specialized innate and adaptive immunologic mechanisms intrinsic to the OS that help regulate the colonization and replication of microorganisms. The stratified corneal epithelium features tight-junctions and desmosomes analogous to other epithelial surfaces. It also expresses membrane-associated mucins MUC1, MUC4 and MUC16, forming the glycocalyx, where MUC16 is thought to be non-permissive to bacterial invasion [4, 5]. The conjunctival epithelium expresses higher levels of MUC4 than the cornea and also harbors goblet cells that secrete the gel-forming MUC5AC, creating a viscoelastic layer that contributes to protection of the epithelium against microorganisms and dehydration [6, 7].

The tear film adds to this antimicrobial defense with its antimicrobial peptides, including lysozyme, alpha and beta-defensins, and secretory IgA [8, 9]. Frequent mechanical sweeping of the OS by the eyelids allows the tear film to trap and remove antigens and unattached microorganisms from the OS [10]. This collection of loosely attached microorganisms eventually is deposited into the dependent conjunctival fornix. This principle has likely in part guided sampling in studies aiming to characterize the OS microbiome using high-throughput sequencing [11, 12]. In addition, collection of conjunctival swabs is associated with minimal discomfort, is not invasive, and does not interfere with corneal integrity. However, based on the OS defense mechanisms discussed above, it is possible that sampling the conjunctival fornix inadvertently includes transient bacteria that do not truly reflect the OS-associated core microbiota. If the cornea is associated with a significantly different microbiome compared to the conjunctiva and tears, this finding could have implications for what constitutes a healthy cornea, and what microbiome profiles are associated with disease states. Differences in microbiome composition have been demonstrated for differing areas of the conjunctiva, and therefore in this study, we hypothesized that the corneal surface harbours a bacterial community different from surrounding OS structures, including the conjunctival fornix and tear film.

## Materials and methods

### Animals

Fifteen crossbred healthy piglets between 10–14 days of age were humanely euthanized for reasons unrelated to this study. Piglets were housed in a biosafety level 2 facility, belonged to a different research group that had to cancel their study thereby leading to immediate euthanasia. As the present study did not involve the use of live animals, an institutional animal care and use committee protocol was not required. These piglets represented a convenient sample where individuals shared the same environment, diet, and genetics. Using a homogenous population aimed to reduce variability and theoretically aided in contrasting microbiome differences between OS sites, if truly present. Pigs were also chosen for their relative similarity to humans regarding eyes, their use in animal models of ocular disease, and their use in corneal xenotransplantation [13–16].

### Ocular surface sample collection

Sample collection was performed in a negative pressure room at a biosafety level 2 facility. All experimenters were gloved, masked, and gowned throughout the sample collection procedure. Both eyes from each pig were sampled immediately following euthanasia. This approach facilitated the avoidance of topical anesthetics when sampling the cornea, a known carrier of contaminants and disrupter of bacterial community profiles [17]. For each eye (*n* = 30), a veterinary ophthalmologist using sterile gloves collected tears by placing a sterile Schirmer tear test (STT) strip (TearFlo Schirmer test strip, HUB Pharmaceuticals, Plymouth, MI) within the ventral conjunctival fornix for 1 minute. Immediately following this, three anatomical sites of the OS (central cornea, inferonasal fornix and superotemporal fornix conjunctiva) were sampled by pressing a quick-drying swab (GenoTube Livestock; Prionics, Switzerland) against the site and performing one continuous circular movement, exposing the entire sampling surface of the swab to the surface in question. Following collection, all samples were immediately brought to and handled inside a biosafety cabinet by a microbiologist, where they were individually stored in sterile 2 mL microtubes. Negative controls for all sample types were collected simultaneously; two swabs and two STT strips were positioned in the same environment where pigs were sampled, and then handled similarly to the other samples. All microtubes were stored at −80°C until processing.

### DNA extraction, 16s rRNA amplification and sequencing

Total DNA was extracted from swabs and STT strips using the PowerSoil® Max DNA Kit (Mo Bio, Carlsbad, California, USA) following the manufacturer’s instructions. Blank controls (no sample added, processed routinely, *n* = 4) were included in the extraction process to control for contamination throughout processing. Total DNA from all samples was measured prior to library preparation using spectrophotometry (ND‐1000, NanoDrop Technology, Wilmington, Delaware, USA), fluorometry (PicoGreen dsDNA assay, Life Technologies, Carlsbad, CA, USA) and 16S rRNA qPCR targeting the V3-V4 region. Briefly, amplification of the 16S rRNA gene V3‐V4 hypervariable region used a KAPA HiFidelity Hot Start Polymerase (Kapa Biosystems Inc., Wilmington, MA, USA) and Nextera primers (Meta_V3_F_Nextera: 5′‐CCTACGGGAGGCAGCAG‐3′, Meta_V4_806_R: 5′‐GACTACHVGGGTWTCTAAT‐3′, Integrated DNA Technologies, Coralville, IA, USA). This first round of amplification used the following cycling parameters: one cycle of 95°C for 5 minutes, followed by 20 cycles of 98°C for 20 seconds, 55°C for 15 seconds, and 72°C for 1 minute.

After the first round of amplification, PCR products were diluted 1:100 and 5 uL were used in the second PCR reaction. This second round used indexing primers (F: 5′‐ AATGATACGGCGACCACCGAGATCTACAC[i5]TCGTCGGCAGCGTC‐3′, R: 5′‐ CAAGCAGAAGACGGCATACGAGAT[i7]GTCTCGTGGGCTCGG‐3′) and the following cycling conditions: 1 cycle at 95°C for 5 minutes, followed by 10 cycles of 98°C for 20 seconds, 55°C for 15 seconds, and 72°C for 1 minute. Pooled, size‐selected samples (based on agarose gel electrophoresis) were denatured with NaOH, diluted to 8 pM in Illumina’s HT1 buffer (Illumina, San Diego, CA, USA), spiked with 15% PhiX, and heat denatured at 96°C for 2 minutes immediately prior to loading the machine. The MiSeq 600 (2 × 300 base pairs, bp) cycle v3 kit (Illumina, San Diego, CA, USA) system was used to sequence DNA libraries.

### Data analysis

DNA quantification comparisons were performed using ANOVA followed by Bonferroni *post-hoc* test. Correlation between DNA quantification methods and number of reads per sample was assessed using a two-tailed Spearman’s correlation test. Sequencing data were analyzed using QIIME2 [18]. Data were demultiplexed and barcodes used to produce coupled fastq files for each sample. Raw amplicon reads were filtered by trimming the adapter sequences and truncating forward reads at 275 reverse reads at 218 bp using the Dada2 plug‐in [19]. Filtered reads were dereplicated and denoised using parameters estimated for this dataset, based on base-calling scores. Next, paired sequences were merged using a minimum overlap of 20 bp and 0 mismatches were allowed. Blank control and negative control samples were used to remove contaminator sequences from all samples through Decontam [20]. Representative sequences were classified against the SILVA SSU Ref NR dataset v.128 at 99% sequence similarity using a classifier algorithm trained for this work’s dataset [21]. Next, contaminant sequences (non‐bacterial, mitochondrial or chloroplast DNA) and sequences not classified beyond the kingdom level were removed from the dataset using the quality control plug‐in within QIIME2. Data retained for downstream analyses were present in at least 10% of all samples, with a minimum of 3 reads/sample. Rarefaction was performed at the smallest library size (*n* = 1104 reads). Differences in alpha diversity (observed, Shannon and Simpson indices) were determined using Friedman’s test, followed by Dunn’s multiple comparison *post-hoc* tests. Beta diversity (Bray-Curtis dissimilarity) comparisons were conducted using PERMDISP and PERMANOVA. Differences in taxa abundance were determined using Friedman’s test, followed by Dunn’s multiple comparison tests. False discovery ratio (log difference > 2-fold) correction was applied to the calculated P-values. Differences were considered significant at P and Q values <0.05. Differential taxa proportion was assessed using DESeq2 on the normalized, filtered dataset [22]. Statistical differences persisted when outliers shown in Fig 5 were removed from the analysis.

## Results

### DNA quantification

**Fig 1:**
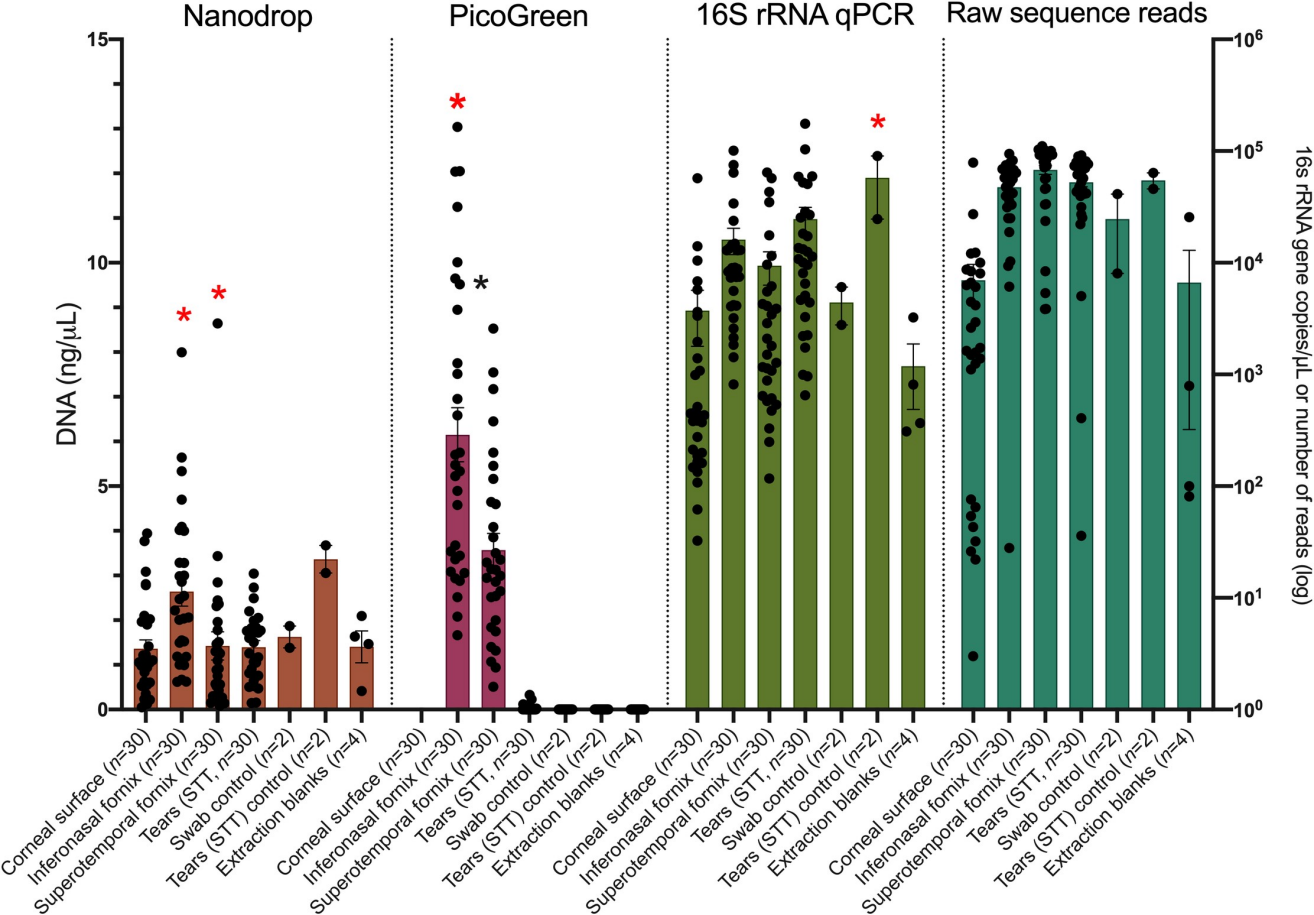
Amount of DNA per sample type according to different measurement methods and resulting raw reads (prior to removal of contaminants) yield. Stars denote statistically significant differences between a given sample type within each methodology (ANOVA followed by Bonferroni *post-hoc* test, *P*<0.05).

A total of 120 samples (30/sampling method) were obtained from 30 eyes, in addition to 2 swabs and 2 STT strips serving as negative controls, and 4 blank DNA extraction controls. Spectrophotometry, fluorometry and qPCR were applied to quantify the DNA load in each sample. While all samples had detectable amounts of DNA using the least specific method (spectrophotometry), corneal swabs and all the control samples yielded no measurable DNA when analyzed by fluorometry. Based on spectrophotometry, the inferonasal fornix yielded the highest amount of DNA (*P*<0.05), followed by the superotemporal fornix (*P*<0.05), and the central cornea and STT strips yielded the lowest amount (no significant difference between the latter two sampling methods). The same pattern was found when samples were analyzed by fluorometry. Surprisingly, STT strip samples yielded the highest average amount of 16S rRNA gene copies among all sample types (*P*<0.05), and no differences were found across the other sampling methods. Negative control swabs and the extraction blank samples also yielded detectable amounts of 16S rRNA gene copies through qPCR. A summary of our findings is shown in Fig 1.

### Amplicon sequencing

A total of 5.4 x 10^6^ high-quality reads from 130 samples were retained for analysis. The median number of sequences per sample was 41.601 (ranging from 3 to 110.722). The number of reads per sample was only positively correlated with the pre-sequencing qPCR measurements (0.42, *P*<0.01). Out of the 5,092 taxa (features) initially detected, removal of contaminating and low-prevalence (<3 reads, present in at least 10% of all samples) sequences resulted in 209 taxa within 109 samples (>1103 reads/sample) being included in downstream analyses.

**Fig 2:**
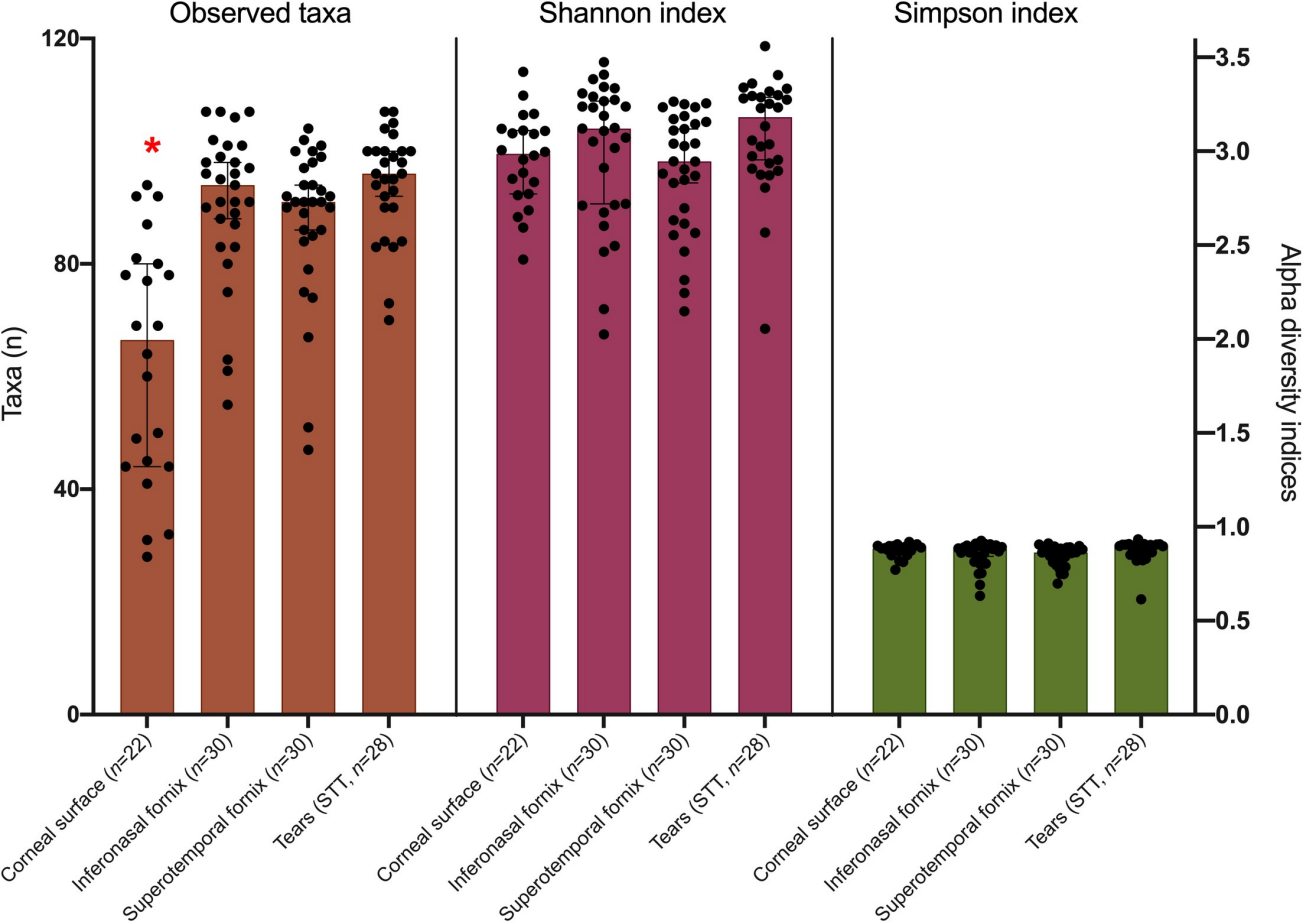
Alpha diversity metrics associated with sampling method investigated, including sample negatives and extraction controls. Star denotes statistically significant differences between sample types within each diversity measurement (Friedman’s test, followed by Dunn’s multiple comparison *post-hoc* tests, *P*<0.05).

Alpha diversity analyses revealed a statistically significant difference between the sampling methods on the number of taxa detected, where corneal samples had the lowest number of taxa detected (*P*<0.01). No difference between the four sampling methods was found when comparing Shannon’s or Simpson’s index (Fig 2).

**Fig 3:**
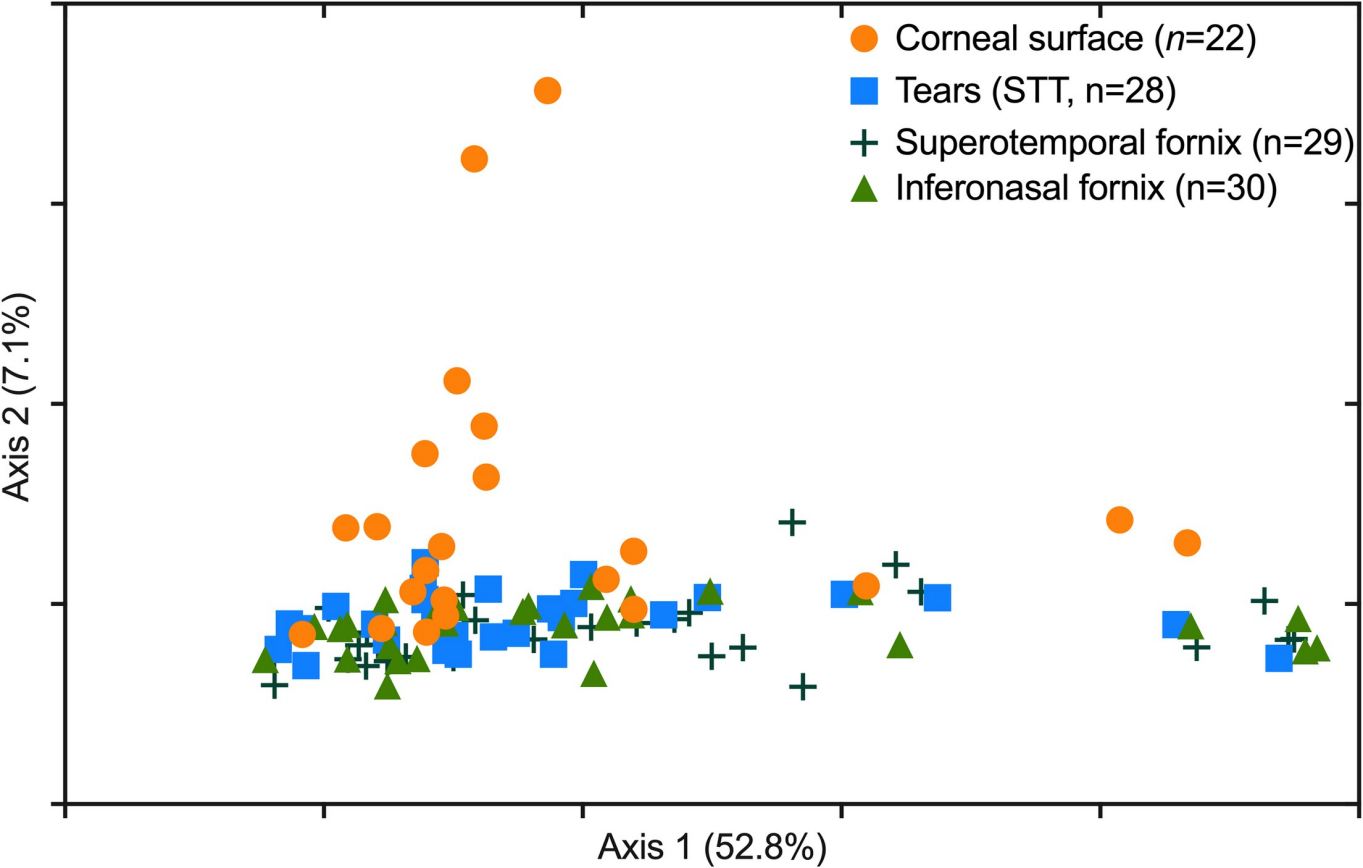
Principal coordinate analysis based on Bray-Curtis dissimilarity indices from each sampling collection method. Corneal samples significantly differed from all other groups (PERMDISP followed by PERMANOVA, *P*<0.05).

**Fig 4:**
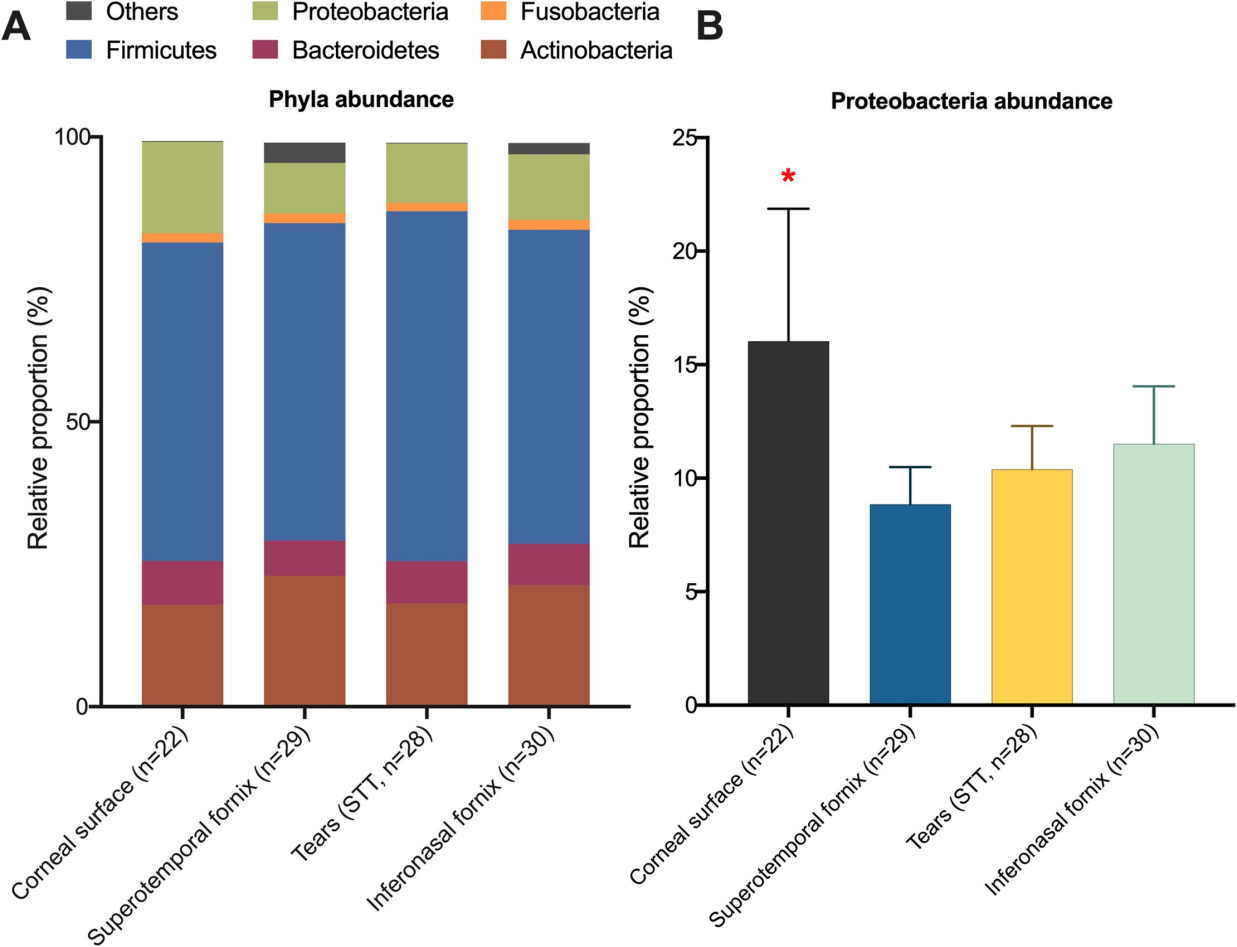
Relative proportion of reads associated with each sampling method at the phylum level (**A**). Average proportion of Proteobacteria within each sampling method (**B**). Star denotes statistically significant differences between sampling methods (Friedman’s test, followed by Dunn’s multiple comparison tests, *P*<0.05).

**Fig 5:**
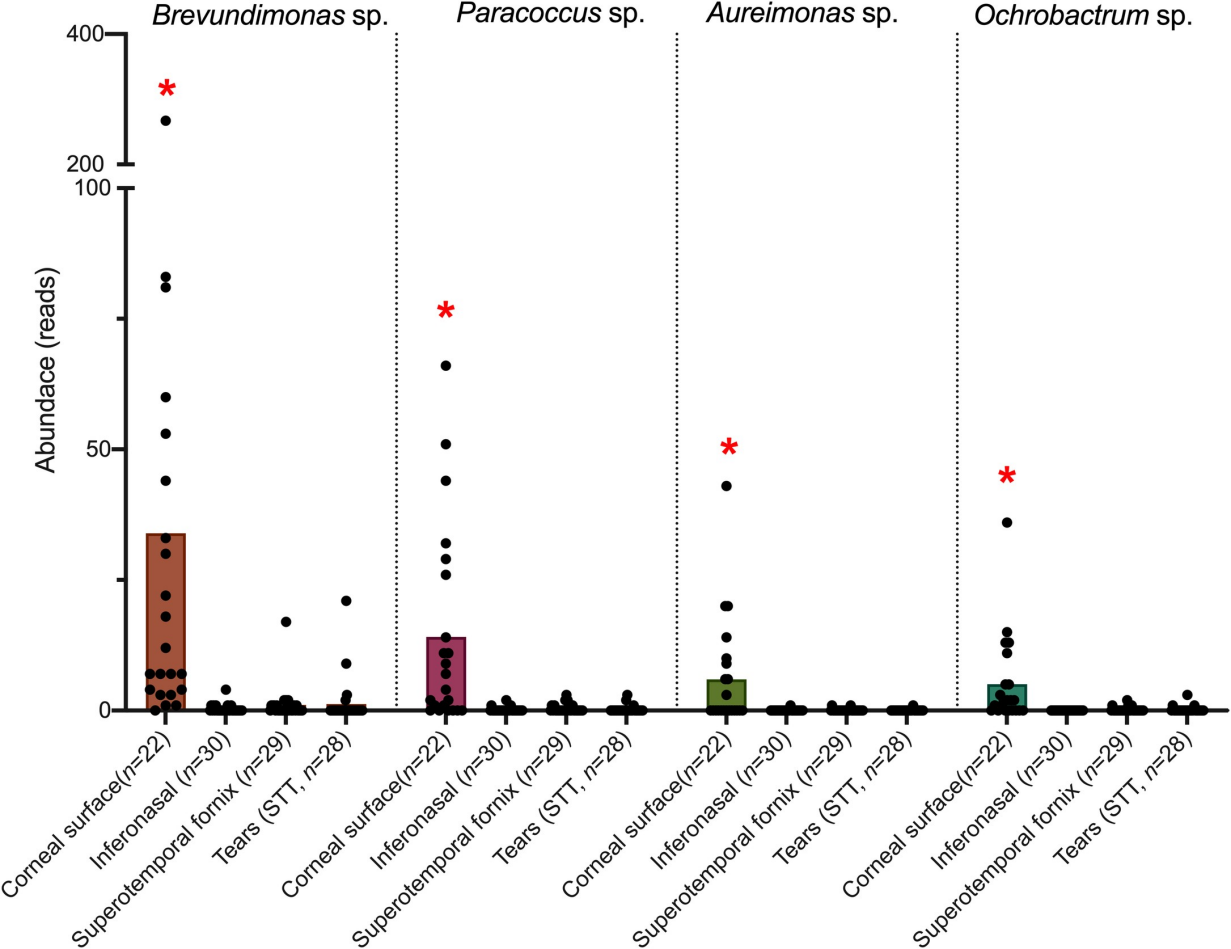
Abundance of reads associated with differentially enriched genera. Star denotes statistically significant differences between sample types within each genus (Q<0.05).

**Fig 6:**
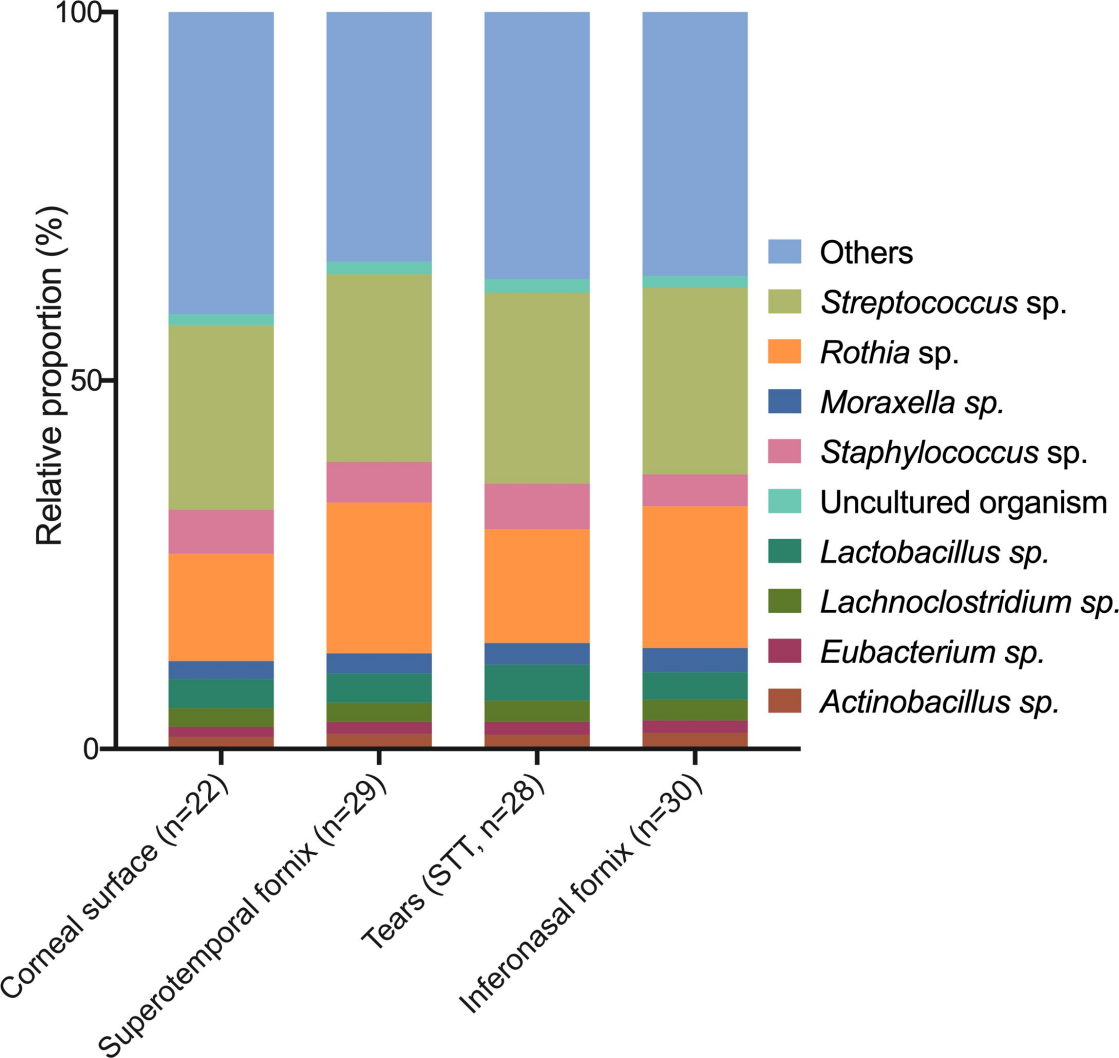
Relative proportion of reads associated with each sampling method at the genus level. Only the top 10 most abundant genera are shown.

Interestingly, comparing Bray-Curtis dissimilarity (beta-diversity) across all sampling methods indicated a difference in community composition between samples (*P* = 0.04). *Post-hoc* comparisons revealed that corneal samples differed from all sampling methods (central cornea vs inferonasal fornix, *P* = 0.01; central cornea vs superotemporal fornix, *P* = 0.02; central cornea vs STT strips, *P* = 0.03; Fig 3). Relative community composition across the different sampling methods is shown in Fig 4A. At the phylum level, Proteobacteria were enriched by 0.9-fold in corneal samples, when compared to the other sampling methods (FDR<0.001, Fig 4B). This observation translated to abundance differences at the genus level, where corneal samples were significantly enriched (FDR<0.001) for *Brevundimonas* sp. (4.1-fold), *Paracoccus* sp. (3.4-fold), *Aureimonas* sp. (2.6-fold) and *Ochrobactrum* sp. (2.3-fold, Fig 5), all of which are members of the Proteobacteria phylum. Also, at the genus level, *Streptococcus* sp. and *Rothia* sp. together accounted for an average of 46% of the reads across all samples, regardless of the anatomical site (Fig 6).

## Discussion

We sampled the OS of 30 healthy piglets to characterize their associated bacterial communities using four methods: swabs of the central cornea, the inferonasal conjunctival fornix, the superotemporal conjunctival fornix, and STT strips. Conjunctival sites are the most commonly used to characterize the OS microbiome using high-throughput sequencing. Our data suggests that fewer taxa are associated with the central cornea than the other sites, and that the bacterial community associated with the corneal surface was compositionally different from the other sites at the phylum and genus level. The bacterial community residing on the cornea is different from the remaining OS.

The OS of mammalian species is comprised of the cornea, conjunctiva and tear film, where the cornea and tear film together are permissive to light [23]. The conjunctiva was the first component of the OS sampled in the era of culture-independent microbiota profiling [3]. Since then, the conjunctiva has remained the most commonly sampled OS site in similar investigations, largely due to ease and safety of sample collection [24–26]. The tear film, despite its intimate contact with the corneal surface, has been scarcely explored as a host of bacterial communities using culture-independent methods [27]. To the best of our knowledge, direct sampling of the corneal surface has not yet been used as a source of DNA for high-throughput sequencing and bacterial metagenomic analysis. While this methodology does not provide evidence of live microbes, the data presented here suggest that the corneal surface harbours a specific bacterial community characterized by a higher proportion of Proteobacteria than the other OS sites (Fig 4). This is congruent with the notion that varying OS sites, such as the conjunctiva, limbus, and fornix have been shown to harbour distinct bacterial communities [17, 28–30]. Each OS tissue is associated with unique growth conditions for colonizing and replicating bacteria, which in turn are linked to differing metabolomic profiles [31, 32]. Thus, it would seem that certain bacteria thrive in certain protected niches which helps to explain the difference in relative composition observed in our study. Our data also revealed that the corneal surface bacterial community was characterized by a reduced number of observed taxa in comparison to the other sampled sites, which again may support the notion of a discrete, specialized environment housing these taxa. However, a difference in the number of taxa was not reflected in the alpha-diversity indices. This is likely due to the fact that Simpson and Shannon’s metrics are based on phylogenetic diversity, while the number of taxa is based on taxonomic diversity [33]. The taxa detected on the inferonasal and superotempral conjunctiva, and the STT strips, but not found on the cornea, could either be transient, washed off from the cornea, truly adapted to those sites, or a combination of these (Fig 2).

The presence of Proteobacteria in swine OS samples was expected, as sequence reads associated with this phylum were consistently detected in high proportions within samples collected from different species, including humans, cats, horses, dogs, marsupials, and birds [24–26, 29, 34–38]. At the genus level, OS studies in humans have reported *Corynebacterium* sp. and *Staphylococcus* sp. as the most prevalent genera [24, 28, 30, 39–42], which are not part of the Proteobacteria phylum. Similarly, our data revealed *Streptococci* and *Rothia* sp., also non-Proteobacteria, as the most prevalent genera of the OS, regardless of sample type. *Streptococcus suis* (the main *Streptoccocci* identified in our study) is a commensal of the upper respiratory tract in swine, however can also be an opportunistic pathogen associated with pneumonia and meningitis in young pigs [43]. *Rothia* spp. are associated with the normal upper respiratory tract of humans and pigs, and have also been associated with caries and periodontal disease in humans [44, 45]. Formation of biofilms is one of the main virulence elements enabling *Rothia* spp., [45–47] which may play a role regarding its survival in the environment of the OS.

Central corneal samples had a higher proportion of Proteobacteria compared to the other OS sampling sites, similar to a study where conjunctival swabs collected with firm pressure yielded a higher proportion of Proteobacteria taxa than conjunctival swabs collected softly [3]. The pressure applied in the current study during corneal sampling was subjectively equal to or less than the pressure during conjunctival sampling, suggesting that the higher proportion of corneal Proteobacteria is indeed a significant finding. Within the Proteobacteria phylum, there were particularly high number of reads associated with the genera *Brevundimonas* spp. and *Paracoccus* spp. *Brevundimonas* are motile, aerobic gram negative rods isolated from multiple environments, including condensation water from a Russian space laboratory [48, 49]. They were also isolated from human clinical samples, including the ocular surface and the central nervous system, which resulted in its status of an emerging opportunistic pathogen [50–52]. Although there are no studies showing that *Brevundimonas* spp. directly attach to mammalian cells, they are capable of co-aggregating with genetically distinct bacteria to form biofilms and have been associated with endocarditis in humans [51, 53]. Similarly, *Paracoccus* spp. are also aerobic, gram negative coccobacilli found in a variety of environments [54]. Bacteria from this genus harbour different virulence factors, including genes associated with host invasion and immune system evasion [55]. Of note, *P*. *yeei* has been previously associated with corneal graft failure following penetrating keratoplasty as well as with traumatic keratitis, further evidencing its adaptation to the OS [56–58]. The limited available literature, together with our findings, suggests that both genera (*Brevundimonas* spp. and *Paracoccus* spp) may have the ability to attach and colonize mammalian corneal cells, possibly in association with biofilms or other mechanisms that would enable their persistence on the OS. Further research to clarify this colonization mechanism is warranted.

There are several possible explanations for why the corneal microbiome could be distinct from the rest of the OS microbiome ranging from its reduced temperature, its anatomic position on the globe, its absence of vascularization, its absence of lymphoid tissue (CALT), differences in MUC protein expression, among other unique features [59]. The clinical implications of a unique corneal microbiome that is distinct from the rest of the OS also remain speculative. If a distinct human corneal microbiome existed, this information could help to broaden our understanding surrounding underlying pathogeneses and immune responses in their contribution to keratitis, both infectious and non-infectious. For example, OS inflammation is recognized to be a major contributor in the pathogenesis of dry eye disease, and its associated microbiome profile has also been suggested to also be a hallmark of disease[42, 60, 61]. Defining the composition of the corneal microbiome in both healthy and diseased states could have clinical implications in understanding corneal disease, and eventually even therapies and prognostication.

The OS microbiome has been previously assumed to be and described as paucibacterial [2] and our data further supports that observation (Fig 1). This further underscores the importance of minimizing contamination during sample collection and the rigorous inclusion of negative controls to prevent nucleic acids contaminants when studying the OS microbiome. Here, we demonstrated that sample controls and extraction controls had very low amounts of DNA detected (or below the level of detection) by fluorometry and spectrophotometry, similar to samples collected from the cornea and tear film. Particularly regarding spectrophotometry, it is noteworthy that, despite the low level of DNA detection, samples had amplifiable amounts of the 16S rRNA gene and yielded high-quality sequencing reads suggesting that extreme caution should be taken when processing OS samples. Surprisingly, the STT strip negative control samples had the highest average load of 16S rRNA gene copies detected across all sample types, despite being marketed as being sterile. STT strips are sterilized using ethylene oxide, which binds to the protein, DNA and RNA of microorganisms and prevents normal cellular metabolism [62]. It does not destroy nucleic acids, which may help explain the high level of 16S rRNA detected in the STT control samples. A similar finding has been previously reported regarding glucose meter test strips used in hospitals [63], but not in commercial STT strips. While this is bacterial DNA most likely originated from non-viable bacterial cells, the presence of bacterial DNA alone can activate pro-inflammatory pathways through toll-like receptors such as TLR-9 [64]. In addition to the inclusion of different levels of negative controls (S1 Fig), samples in this study were collected immediately post-mortem, and therefore enabled an anesthetic-free approach thereby eliminating contamination and significant alteration of community structure and composition associated with topical ophthalmic anesthetics [17]. We also applied a post-sequencing approach to identifying contaminants using the Decontam package, which highlights differences in sequence prevalence and/or frequency between real and control samples to identify contaminants [20]. This approach has been suggested to be more effective in the control for contaminants than the total exclusion method [65].

A homogenous population of pigs was used in this study which was important for bringing to light microbiome differences as a function of OS sites. Evidently, humans differ dramatically in their diet, environment, and genetics, and therefore our findings should be further validated using human samples.

This proof-of-concept study sheds light on the existence of particular microbial niches within the ocular surface. Further investigations are needed given that the methodologies used here (amplicon high-throughput sequencing) only reflect the presence of genetic material, which is not necessarily associated with viable microorganisms. In addition since this was not a longitudinal study, the data generated does not allow for interpretations regarding the stability of the putative cornea-dedicated bacterial community.

Here, we have used pigs as a biomedical model in this proof-of-concept investigation due to their physiological and anatomical similarities to humans [66]. To the best of our knowledge, this is the first study to show that the corneal surface bacterial microbiome is compositionally different from other ocular surface structures. While further evidence of this difference is required to support this claim, and other studies are in progress to provide this, our observations may have a significant impact on the current understanding of the OS microbiota in both healthy and diseased states as well as immunological homeostasis.

## Supporting information

S1 Fig Relative proportion of reads associated with individual control samples and the average for each sampling site at the phylum level. Controls were significantly different from sampled sites based on Bray-Curtis dissimilarity (PERMDISP followed by PERMANOVA, *P*<0.05) and Shannon’s diversity index (Friedman’s test, followed by Dunn’s multiple comparison *post-hoc* tests, *P*<0.05).
(TIF)
