# Supplementary figures and images for: The porcine corneal surface bacterial microbiome: A distinctive niche within the ocular surface
Source: PLoS One. 2021 Feb 19;16(2):e0247392. doi: 10.1371/journal.pone.0247392 (PMC7895408; doi:10.1371/journal.pone.0247392)

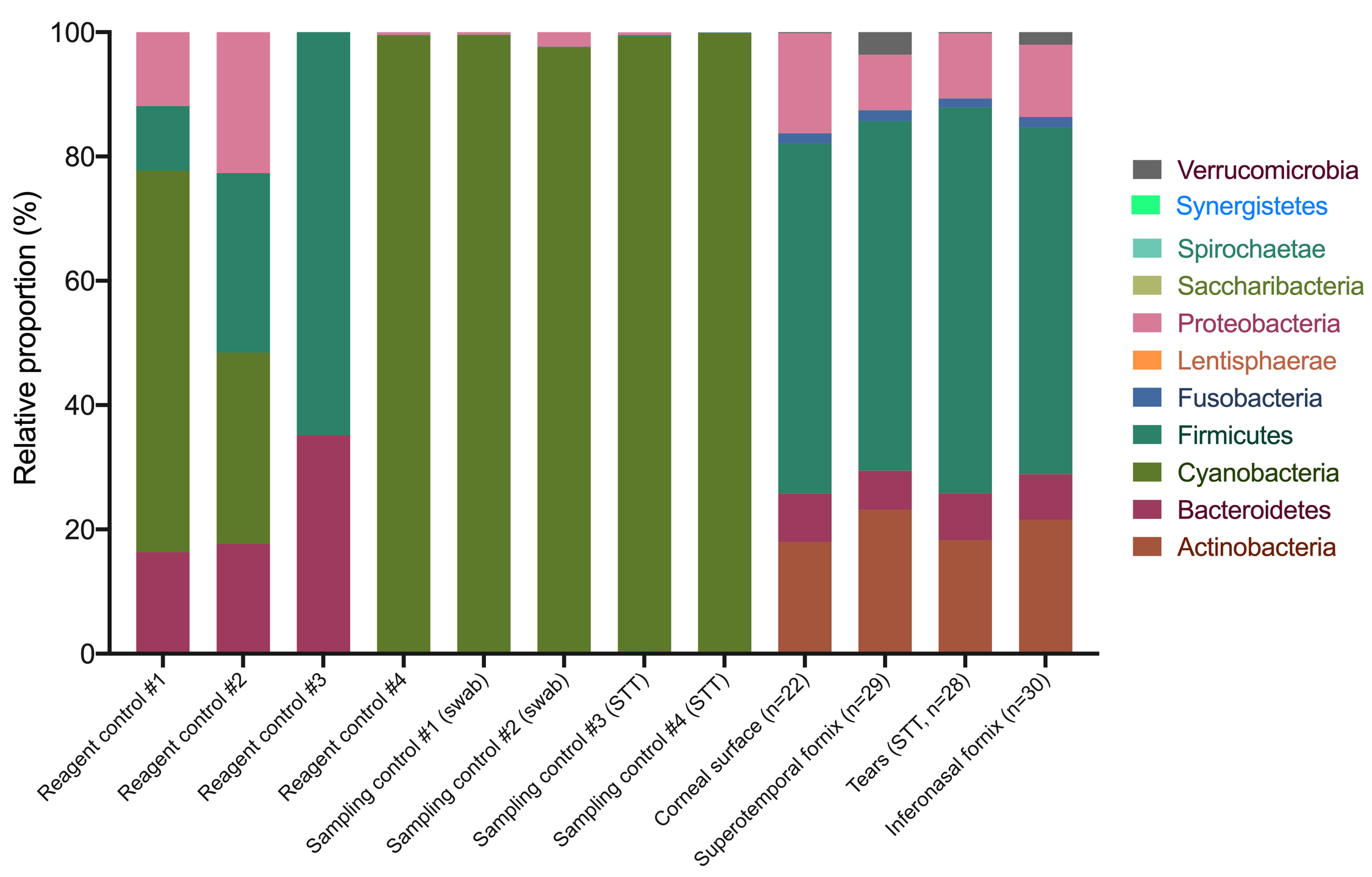

Supplement: S1 Fig — Controls were significantly different from sampled sites based on Bray-Curtis dissimilarity (PERMDISP followed by PERMANOVA, P<0.05) and Shannon’s diversity index (Friedman’s test, followed by Dunn’s multiple comparison post-hoc tests, P<0.05). (TIF) [file pone.0247392.s001.tif]
